# Risk, benefit, and social value in Covid-19 human challenge studies: pandemic decision making in historical context

**DOI:** 10.1007/s40592-022-00156-6

**Published:** 2022-06-15

**Authors:** Mabel Rosenheck

**Affiliations:** Independent Scholar, 424 Morris Street, #2, 19148 PA Philadelphia, USA

**Keywords:** Covid-19 pandemic, Human challenge studies, Medical Research, Research Ethics, Social Value, Vaccines

## Abstract

During the Covid-19 pandemic, ethicists and researchers proposed human challenge studies as a way to speed development of a vaccine that could prevent disease and end the global public health crisis. The risks to healthy volunteers of being deliberately infected with a deadly and novel pathogen were not low, but the benefits could have been immense. This essay is a history of the three major efforts to set up a challenge model and run challenge studies in 2020 and 2021. The pharmaceutical company Johnson and Johnson, the National Institutes of Health in the United States, and a private-public partnership of industry, university, and government partners in Britain all undertook preparations. The United Kingdom’s consortium began their Human Challenge Programme in March of 2021.

Beyond documenting each effort, the essay puts these scientific and ethical debates in dialogue with the social, epidemiological, and institutional conditions of the pandemic as well as the commercial, intellectual, and political systems in which medical research and Covid-19 challenge studies operated. It shows how different institutions understood risk, benefit, and social value depending on their specific contexts. Ultimately the example of Covid-19 challenge studies highlights the constructedness of such assessments and reveals the utility of deconstructing them retrospectively so as to better understand the interplay of medical research and research ethics with larger social systems and historical contexts.

**Supplementary information:**

The online version contains supplementary material available at 10.1007/s40592-022-00156-6.

## Introduction

In March of 2021 at the Royal Free Hospital in London, Jacob Hopkins and Alastair Fraser-Urquhart were among the first volunteers to have SARS-CoV-2, the virus that causes Covid-19, dripped into their noses on purpose (Grover 2021, Fraser-Urquhart 2021). They were part of the world’s first Covid-19 human challenge study. In such a study, healthy volunteers like Jacob and Alastair are deliberately infected with a pathogen for research purposes. In doing this, scientists can better study the human immune response, and they can rapidly test vaccines, treatments, and other interventions against disease. The advantages of speed, laboratory conditions, close observation, and precise data collection make controlled human infection a unique tool in medical research, especially vaccine research. They are a tool that researchers have used for centuries, at least since 1796 when Edward Jenner tested his smallpox vaccine by inoculating a nine-year-old boy named James Phipps and then exposing him to the virus (Baron 1827). These studies may defy the tradition of the Hippocratic Oath and its simplistic principle that physicians and scientists should never do harm to their patients or their research subjects. However, in the modern context, the immense benefit to huge numbers of people that can come from such harm can make the seemingly utilitarian rationale compelling. Matthew Memoli, a leading challenge study researcher at the National Institutes of Health (NIH) in the United States, explained his work this way: ‘I’m not there to make them better… I am there for the benefit of society and the world to generate data that can make everyone better in the long run’ (Zaleski 2020).

In 2020 and 2021, many ethicists, researchers, vaccine developers, funders, and potential volunteers considered challenge studies for Covid-19. The theory was that by risking the health of a few hundred volunteers, thousands if not hundreds of thousands of lives could be saved from the global pandemic. The first proposals came in March of 2020. Further discussion followed in April and May. The idea was debated through spring and summer. Preparations continued through fall and winter. By January of 2021, most had lost interest, but researchers in the UK persisted. They inoculated the first volunteers a year after the pandemic began.

Over the course of the year, supporters and opponents made arguments for and against challenge studies in terms of a dozen or more categories of risk, benefit, justice, volunteer autonomy, and feasibility. By the end of 2020, the literature on the ethics of Covid-19 challenge studies was large (Eyal et al. 2020, Plotkin and Caplan 2020, *Key criteria for the ethical acceptability of COVID-19 human challenge studies* 2020, Shah et al. 2020, Jamrozik and Selgelid 2020, Schaefer et al. 2020, Dawson et al. 2020, Elliott 2020, Kahn et al. 2020). The literature on the science of Covid-19 challenge studies was growing larger (Nguyen et al. 2020, Deming et al. 2020, Lambkin-Williams and DeVincenzo 2020, Douglas and Hill 2020). Throughout 2020, leadership in the UK government and at the NIH in the United States were also taking the idea, with all its risks and benefits, seriously. Major pharmaceutical companies and major philanthropies gave thought to the idea too. In the end, it was a British consortium, the NIH, and Johnson and Johnson Pharmaceuticals in collaboration with Leiden University Medical Center in the Netherlands that began preparations. Each went so far as to manufacture challenge strains and develop a research protocol, but it was only the British that ran challenge studies during the pandemic. The scientific potential and the social value of this research provided reasons to run Covid-19 challenge studies, but there were drawbacks, practicalities, and risks–both individual and societal–that complicated the benefits, however great they might have seemed at first glance.

This essay draws on critical theory and approaches from science and technology studies to put medical research and ethical debates in dialogue with the social, epidemiological, and institutional conditions of the pandemic as well as the commercial, intellectual, and political systems in which medical research and Covid-19 challenge studies operated. This approach encourages further exploration of the differences in how each institution decided whether or not to run challenge studies and their distinct constructions of risk, benefit, and social value. Key to my discussion is the fact that these decisions involved the consideration of science and ethics, but they were also socially and institutionally constructed. The decision making was not natural. The risks and benefits were not self-evident or universally understood in the same ways. Researchers and administrators examined the conditions under which they might or might not take on these risks. They then made choices that were informed by science and ethical principles, but were also contingent on historically and institutionally situated interests and contexts beyond them. Covid-19 challenge studies are a useful case study because a range of institutions pursued them and each calculated risk and benefit differently. This essay will proceed through the story of each effort focusing on the question of risks and benefits. It will conclude with a broader discussion of what was and was not considered with regards to social value and vaccine equity. Ultimately the example of Covid-19 challenge studies highlights the constructedness of such assessments and reveals the utility of deconstructing them retrospectively so as to better understand the interplay of medical research and research ethics with larger social systems and historical contexts.

### Literature on Covid-19 Challenge Studies

Though the idea of deliberately infecting people with a virus like SARS-CoV-2 was startling for many, it didn’t come out of nowhere. As early as February and March of 2020, there were several bioethicists and scientists thinking about such an undertaking. Among the scientists were Matthew Memoli at the NIH, Meta Roestenberg at Leiden University in the Netherlands, and Garth Rapeport at Imperial College. They had all been running challenge studies regularly for over a decade and were part of the growth and mainstreaming of the approach (Roestenberg et al. 2018). Because of this background, they recognized early their potential use for Covid-19. In the field of bioethics, Seema Shah, Holly Fernandez Lynch, Franklin Miller, Charles Weijer, Michael Selgelid, and Euzebiusz Jamrozik were among those with existing expertise in the ethics of challenge studies who added their voices to the discussion. Stanley Plotkin, Arthur Caplan, Nir Eyal, and Marc Lipsitch, the scholars who initiated the public debate in respective publications, brought expertise in vaccines and vaccine trial design from varied backgrounds in scientific research, philosophy, and epidemiology.

When these and other experts looked at the crisis that was emerging rapidly in the early months of 2020, they saw the looming devastation. They also saw that vaccines would be a key solution in fighting the pandemic. Challenge studies might then have a role to play in their development. Under the right conditions, challenge studies for Covid-19 could be ethical if the risks were minimized enough and the benefit was maximized enough. Ethicists and scientists varied in their certainty that those conditions could be met, but supporters easily imagined situations that might produce the appropriate balance (Eyal et al. 2020, Eyal 2020, Eyal 2021, Caplan 2021, Plotkin 2021).

The case for challenge studies hinged largely on anticipated social value, though the reality of the risks and benefits were more complicated. The core of the argument made by supporters was that challenge studies could speed vaccine development by a matter of months and save tens of thousands of lives (Eyal et al. 2020, Caplan and Plotkin 2020). Eyal et al. were most optimistic. They suggested that a challenge study might supplement or even replace a phase three trial and accelerate the path to licensure by eliminating or reducing the lengthy process of recruiting and running a study that would require tens of thousands of participants waiting to be infected by chance. They argued that having a vaccine authorized sooner would have immense social value by improving health, saving lives, and producing scientific knowledge. Others emphasized that getting a vaccine sooner could return society to normal more quickly and minimize the economic devastation of the pandemic (Yglesias 2020, Tabarrok 2021). In these ways, a challenge study might have society-wide impact and immense social value, even if it lacked direct benefit for the individual volunteer.

More realistically, challenge studies could have been used as a supplement, rather than a replacement, to various phases of the vaccine development process. By generating early efficacy data, they could have been used to quickly downselect from the dozens of vaccine candidates in development. Only the most promising would then get the large-scale investment of a phase three trial. Having early efficacy data could also have helped pharmaceutical companies make decisions about when to scale up manufacturing so as to have doses ready once they were approved, however they were approved. Further, challenge studies could have been used to understand the disease better. They might have been used to find correlates of protection, to study the immune response broadly, and to gather information on aspects of the virus like its transmission that could inform public health policy. However, the broadest consensus among scholars and scientists was that if the virus was no longer circulating at high levels by the time phase one and two vaccine trials were completed, it might be impossible to get results from a standard field trial and a challenge study would be the best, and perhaps the only, path to a vaccine and out of the pandemic (Nguyen et al. 2020, Shah et al. 2020, Jamrozik and Selgelid 2020). This opinion was even shared by those otherwise skeptical of challenge studies for Covid-19 (Deming et al. 2020, Bramble 2021).

Of course, the benefits were accompanied by risks. Most of these were established early in the pandemic and became clearer over the succeeding months. There was no denying that this was a deadly pathogen. However, by the end of March, there was good data from the outbreaks in China that showed that young people had only a 0.03% mortality rate and a 1% rate of hospitalization (Verity et al. 2020). It was also becoming clear that co-morbidities like heart and lung disease, diabetes, and obesity enhanced susceptibility to hospitalization and death. This meant that by selecting young, healthy adults, the risk to volunteers could be quite low. By the time the team at Imperial College submitted the protocol for their challenge study for review, data showed that the actual risk of death for a young, healthy volunteer was 1 in 250,000 (Rapeport et al. 2021). Over the course of 2020, researchers came to better understand how low the risk of death was because of more and better data. In addition, some antiviral and anti-inflammatory therapies were found to be effective. Just as importantly, as standards of care improved, the risk of death further decreased even without the reliable pharmaceutical interventions or “rescue therapies” that many called for. On the other hand, while the known mortality risk decreased over time, the known risk of long term impacts increased as doctors and patients came to understand more about Long Covid. The lasting effects of Covid-19 gave credence to the argument that doctors simply didn’t know enough about the disease to deliberately infect people with it.

There were other concerns that emerged too. These were not necessarily specific to Covid-19 challenge studies, but they were highlighted in the literature and the public discourse as particularly salient for this research in this context. The first concern was that developing a challenge model–characterizing the virus, manufacturing a strain using good manufacturing practices (GMP), finding appropriate facilities, getting approvals from regulators and ethics committees, and conducting the initial dosing study–could take anywhere from a few months (an optimistic estimate made by Plotkin and Caplan (2020)) to a year or more (an estimate based on non-pandemic levels of urgency, speed, and collaboration made by Deming et al. (2020)). There were concerns about redirecting resources including hospital facilities, personal protective equipment, and staff (Memoli 2021a, Kahn et al. 2020). There were questions about the generalizability of a study conducted only on young, healthy people. There were questions as to whether regulatory agencies would consider such limited data for licensure (Corey et al. 2020, Science Weekly 2020, Khan et al. 2020).

The broader risk to public trust, not just to the health of the individual, was more difficult to quantify, but it also may have been the most consequential. The scale of possible impact on society writ large–even if the individual chance of death, hospitalization, or Long Covid was small–meant that an adverse event could, in some sense, be even more harmful to society than to the individual volunteer, making it that much riskier (*Key criteria for the ethical acceptability of COVID-19 human challenge studies* 2020, Kahn et al. 2020). This risk in particular had different currency for the NIH than for Johnson and Johnson and for public versus private institutions.

Finally, there were concerns about consent, exploitation, and the autonomy of the volunteer (Dawson et al. 2020, Elliott 2020, Weijer 2021). Compensation and post-trial health care were issues that sponsors would have to address, especially in the United States where health care, post-trial or otherwise, was rife with inequity when it was available at all (Elliott 2020, Lynch et al. 2021). Finally, the risk to the reputation of those who developed or used a challenge model was a key consideration, though it was not, in so many words, an ethical concern. Indeed, several of these risks were beyond the scope of traditional research ethics. They were nonetheless necessary considerations in the decision making of relevant parties.

In sum, there were a range of risks at issue including risks to the individual volunteer, to society, and to the sponsor. However, many believed that the ceiling on what risk was acceptable was not absolute. Rather, it was dependent on the benefit and the social value of the study which, in turn, was dependent on a range of considerations, some more controllable than others (Shah et al. 2020, Evans 2020, Menikoff 2020). Most obvious was that the quicker a model could be set up, the more useful it would be, and the more lives it could potentially save. In reality, a model wasn’t set up as quickly as supporters hoped and other contexts rapidly overtook the initial benefits laid out in favor of challenge studies.

## Primary efforts

### Pfizer, Moderna, and AstraZeneca

Eyal, Lipsitch, and Smith posted their article as a pre-print on March 24, and it appeared online in the *Journal of Infectious Diseases* on March 31, making it the first academic article published on Covid-19 challenge studies (Lipsitch 2020, Eyal et al. 2020). The proposal to fully replace a phase three trial with a challenge trial, speed up vaccine development by months, and save thousands of lives was a dramatic hook. Coming when it did, it convinced many, even those outside of the medical and bioethics fields, that challenge studies should be pursued (Morrison 2020b).

Importantly, the promise of a challenge study emerged amid a moment of immense uncertainty surrounding the still emerging pandemic and the possible timeline for a vaccine that could end it. As early as February, Anthony Fauci, director of the National Institute of Allergy and Infectious Diseases (NIAID) at the NIH, said a vaccine would be available for use in 12–18 months, but many were skeptical (“Danger of getting coronavirus” 2020). Some, like Arthur Caplan, thought that timeline was unlikely, if not outright impossible (Love 2020). Others, like Eyal, wondered if it could be done even faster (Eyal et al. 2020). Still others worried about what would happen if testing proved difficult because the virus was under control or recruitment of volunteers was slow (Caplan 2021). Another fear was that a vaccine might meet Fauci’s timeline, but still prove ineffective as many vaccines do, returning the process to the beginning. A challenge model might be able to prevent this scenario or speed up the process for a second round of candidates even if not the first. To many, amid the desperation of those first months of the pandemic, challenge studies seemed like a solution tailor made for the urgency and uncertainty of their circumstances. Supporters may have been overly optimistic about the benefits, but at that moment, the risk to young, healthy volunteers seemed reasonable because the benefits and the potential value for all of society seemed enormous. As scholars debated the issue in academic journals, people, on their own, began to volunteer (Friedersdorf 2020, Kleinwaks 2020, Gokey 2020, 1Day Sooner 2021).

For many people who saw the value of a challenge study exclusively in terms of its support for the development of first generation vaccines, their value rapidly declined when the United States established Operation Warp Speed, an initiative to coordinate massive government investment in vaccine development. President Donald Trump along with the Department of Health and Human Services and the Department of Defense formally announced the public-private partnership in May. Already in March and April, the US government’s Biomedical Advanced Research and Development Authority (BARDA) had distributed over $400 million each to Moderna Therapeutics and to Johnson and Johnson for vaccine development and clinical trials. Ultimately, the US federal government spent over $18 billion to fund the testing and manufacturing of vaccine candidates from Moderna, AstraZeneca, Johnson and Johnson, Novavax, and Sanofi/GSK, with Pfizer/BioNTech accepting an advanced market commitment, but no funding for development or testing (Baker and Koons 2020). In other words, Operation Warp Speed both downselected to the first generation of vaccines from a large field of candidates and funded manufacturing so production could begin early. They did both without the guidance of challenge studies. By early summer, the value of two of the primary advantages provided by a challenge study had diminished.

In addition to the funding provided by Operation Warp Speed, coordination, resources, and other support from the federal government further bolstered efforts like Moderna’s and Johnson and Johnson’s. These reources enabled the NIH to effectively coordinate their research network and streamline the process through which they tested the candidates which Operation Warp Speed pre-selected for accelerated development (Corey et al. 2020). Every stage moved far faster than just about anyone could have imagined. Bureaucracies like the Food and Drug Administration (FDA) moved far quicker than usual as well. Under normal conditions, a challenge study–even one that took a year to set up–could have been invaluable in a number of ways. However, the availability of these public resources meant that many of the obstacles which challenge studies could eliminate just weren’t obstacles. In the end, that meant that a standard randomized controlled phase three trial could begin and end sooner than a challenge study could. It could also garner better data for safety, efficacy, and licensure. Already by late spring, positive interim results from Moderna’s phase one and animal studies made people like Fauci optimistic about the vaccine’s efficacy. This and the speed at which the trials were proceeding encouraged the belief that the best and fastest vaccine testing would take place in conventional ways (“Fauci says” 2020, Moderna, Inc. 2020a, Corey et al. 2020). The case that challenge studies had a role to play in developing these first vaccines was quickly becoming less convincing.

On July 27, Pfizer and Moderna began enrollment for their large phase three trials (Pfizer 2020a, Moderna, Inc. 2020b). There was still concern throughout the spring and summer that lockdowns and public health measures would be effective, that the virus would be under control by fall, and that it would be difficult to test vaccines in a phase three trial conducted in the United States. However, as August came to an end, it was clear that disastrous public health responses meant limited circulation of the virus wouldn’t be a problem.^1^

September and October saw cases and death tolls rising across the country and around the world as the next wave of the pandemic emerged. Covid-19 was circulating at increasingly high levels which meant research subjects were more likely to contract the virus in their everyday lives. As a consequence, Pfizer and Moderna were able to announce the results of their trials in November. Their vaccines showed astonishing efficacy rates over 90% (Pfizer 2020b, Moderna, Inc. 2020c). In December, the FDA granted emergency use authorizations to both (Pfizer 2020c, Moderna, Inc. 2020d**)**. In parallel, AstraZeneca announced strong efficacy results on November 23. On December 30, UK regulators authorized the vaccine that was created in partnership with Britain’s own Oxford University (AstraZeneca 2020). This did not exhaust the use cases for challenge studies, as the UK Human Challenge Programme would show. However, for most, it was harder to see a convincing rationale for research into prevention of a disease that a vaccine could now prevent reliably (Rouphael 2021, Shah 2021, Booth and Johnson 2020). The decision made by Moderna, Pfizer, and AstraZeneca not to pursue challenge studies seemed, to most, like a sound one. In the end, challenge studies were unneeded and so it seemed a correct estimation that the benefits to public health would not have outweighed the risks to volunteers.

Of course, these vaccines still could have proved ineffective no matter how fast the testing went. It’s reasonable to ask whether a challenge study could have sped up the 11 month timeline of those first vaccines. It’s also reasonable to wonder what might have happened had those vaccines failed. Even if a challenge study couldn’t speed development of the first vaccines, if those had proved ineffective, it might have aided with the testing of others. The 90% efficacy rates were not expected and they were not the norm. Vaccine development had never gone this fast, with this kind of result, and, further, no pandemic had ever been ended with a vaccine, as it seemed this one might be. This reality might be an argument for why challenge studies should have been prepared with more urgency, but it is also simply a reminder of the particular moment in which the proposals emerged and then took off. The idea didn’t just take off because it was a good one.

### Johnson & Johnson

Not all of the vaccine developers were in the same position as Moderna, Pfizer, and AstraZeneca. In a July 21 hearing held by the United States House Committee on Energy and Commerce, members of congress asked executives from the major pharmaceutical companies about challenge studies. Pfizer and Moderna, whose phase three trials were imminent, indicated that they had no intention to conduct challenge studies (Young 2020, Hoge 2020). However, Johnson and Johnson, represented by Janssen Pharmaceuticals, its Belgian subsidiary, left the door open. Macaya Douoguih, the Head of Clinical Development at Janssen, simply stated: ‘We have not yet made a decision about whether to conduct human challenge studies’ (Douoguih 2020). They hadn’t made a final decision, but Janssen was, in fact, sponsoring the preparation of a challenge strain, and they were doing so for the exact reasons Pfizer and Moderna weren’t.

Johnson and Johnson began preparing their vaccine in January along with dozens of other companies, but they didn’t complete their candidate until March and didn’t begin phase one/two testing until late July (Zimmer 2020). They became concerned that their delayed timeline would impact the feasibility and speed of their trials. More so than other companies, they feared that recruitment would be slow or that there wouldn’t be enough virus circulating to get reliable results quickly once their phase three trial finally began. In response to these concerns, they began discussing challenge studies as an option (Guarascio 2020, Morrison 2021a, Morrison 2021c).^2^ The thinking at Johnson and Johnson reflected the belief held by many ethicists, researchers, and regulatory officials that the benefits of a challenge study would outweigh the risks if a situation arose where vaccines were still needed, but could no longer be tested in conventional ways (Guarascio 2020, Deming et al. 2020, Grady 2020, Food and Drug Administration 2020). Janssen was willing to take a risk that others weren’t, but their logic about the benefits and social value of a challenge study was not out of sync with ethical frameworks produced by scholars, researchers, or the WHO.

By August, preparations for viral manufacture were underway with Janssen funding the production of a GMP strain in collaboration with Leiden University Medical Center in the Netherlands (Morrison 2021b, Morrison 2021c). As preparations for challenge trials proceeded, Janssen started their randomized controlled phase three trial. They began the trial on September 23, 2020 and completed the enrollment of 45,000 volunteers on December 17 without any major delays (Johnson & Johnson 2020a, Johnson & Johnson 2020b). Revealing positive results just 6 weeks later, they filed for an emergency use authorization in the United States on February 4th, 2021 (Johnson & Johnson 2021a). The FDA granted the authorization on February 27th (Johnson & Johnson 2021b). For Johnson and Johnson, the reason to pursue challenge studies was to speed the development of an efficacious, first generation vaccine. However, the trials went quickly and the challenge strain and the dosing study weren’t ready early enough to make a difference. When the fears that outbreaks would wane, vaccines would fail, and challenge studies would be both ethical and necessary weren’t realized, neither were the studies themselves. Less than ethics, it was, once again, efficient trials coordinated by the NIH and a public health response not coordinated at all that made challenge studies unnecessary.

The reasoning behind Johnson and Johnson’s decision was understandable. They reacted logically to logistical and epidemiological conditions that undermined the social value of a challenge study and its unique potential to speed vaccine development and save lives. The viral strain, the dosing study, and the relevant regulatory approvals were not ready before the phase three trial reached its endpoints and authorization was granted. The GMP manufacturing process probably could have gone faster, but still the set up time proved a major limitation to the benefit of a challenge model. Ultimately, if a challenge study wasn’t going to speed authorization, it was not going to be beneficial enough for Johnson and Johnson to pursue it further.

Although there were many additional uses and many ways challenge studies could have helped fight the scourge of Covid-19, those uses wouldn’t have been a priority for Johnson and Johnson as a pharmaceutical company and vaccine developer.^3^ Their priority was commercial development of their candidate, not basic science. This reflected a standing difference between academic researchers and industry product developers that is especially evident in their use of challenge studies. Academic researchers focus acutely on collecting all of the data possible to increase the social and scientific value of their studies and to honor the risk volunteers are taking (Memoli 2021b, Rapeport 2021, Roestenberg 2021). Pharmaceutical companies are likely to be more single-minded in their approach. The range of things a challenge model could help scientists understand–natural history, immunology, transmission–and the range of ways that that understanding could serve science and public health would be peripheral, if not entirely absent, considerations. Of course any safe, effective vaccine would be good for the public. That’s why received hundreds of millions of dollars in public financing. However even with that investment, Johnson and Johnson was unlikely to make its vaccine a true public good. They funded the strain because, if they needed it, they would be able to use the model to test their vaccine, bring it to market, save lives, and also make a profit.^4^ There would have been social value to the world, of course, but the financial value to the company was critical.

If there was a set of universal benefits (accelerated vaccine development) and risks (the health of volunteers) to running a challenge study, there was also a unique set of financial benefits and reputational risks to Johnson and Johnson as one of the world’s largest pharmaceutical corporations. The public might view an altruistic volunteer being hospitalized or even dying in the search for a vaccine as a heroic sacrifice akin to fire fighting or military service, but that vision can sour quickly when the sacrifice is made for an uneasy mix of profit and human well being. That could be as bad for Johnson and Johnson–a company already embroiled in blame for the American opioid crisis–as for public trust in vaccines, medical research, and science.

Johnson and Johnson had reason to take these risks, but navigating those risks with the public would have been a challenge of its own. Scientists, ethicists, and administrators constructed risk and benefit internally, doing so in terms of science and ethics as well as their unique institutional concerns. Just as importantly, however, had they run challenge studies, they would have had to present the risks, benefits, and broader social value for the public too. This would have required the expertise of scientists and ethicists certainly, but public relations professionals would be just as central. Scientists can minimize risk and maximize benefits. Public relations can create a discourse that manages the risk to corporate reputation and to public trust by demonstrating the benefits to society. The concepts of beneficence and social value are ethical ones that must inform decision making on one level. However, in the case of public relations, the narrative of risk and benefit may operate on a second level that negotiates science, ethics, public concerns, and public perceptions in ways that take into account social and institutional contexts alongside principles and protocols. Decision making took place on an internal, administrative level, but it is here in corporate public relations that the construction and the constructedness of risk, benefit, and social value is perhaps most literal.

### The National Institutes of Health

Since vaccine developers were the target users of a challenge model, their financial investment, their willingness to take risk, and their view of the benefits was an important set of variables that influenced how challenge studies might be used and whether or not they would be run at all. However, the public discussion around challenge studies in the United States didn’t focus on industry decision making. It focused on the opinions of officials at NIAID and the NIH. Unfortunately for challenge study advocates, NIH leadership was never enthusiastic about the project. In early March when Matthew Memoli first suggested that challenge studies were worth preparing, NIAID director Anthony Fauci and his staff were surprised by the idea. Unlike those with experience in challenge studies, it wasn’t a prospect that had occurred to them organically. ‘They had to sit down and think about it,’ said Memoli, paraphrasing the administration’s reaction to his unexpected proposal (Memoli 2021a). Memoli himself didn’t yet know if the conditions–ethical or otherwise–would be right, but by late February and early March experts like him and others were hearing the alarms louder and louder that this pandemic could be catastrophic and that now might be the time to take a less conventional approach.

Leadership at the NIH hadn’t considered challenge studies as early as some individuals had. As academic and public discussion built through April and May, they were forming a skeptical position. In a May 1st article, Christine Grady, Chief of Bioethics at the NIH Clinical Center, told STAT: ‘I wouldn’t take it off the table, but I certainly wouldn’t say we’re ready for it now… And I certainly wouldn’t let it divert activity from other ways of testing vaccines’ (Branswell 2020). In a May 11 article, Francis Collins, the NIH director, shared his opinion that:

I’m not sure how much it would accelerate the timeline. And of course, the human challenge trials have multiple ethical issues associated with them… I think the weight of evidence is that while this is an interesting conversation to have, at the present time it doesn’t seem like the right path to travel down. (Griffin 2020)

Around the same time, Fauci, Collins, Lawrence Corey, and John Mascola published an academic article that expressed equal skepticism about the generalizability of data and the scientific utility of challenge studies for the broader program of vaccine research and development (Corey et al. 2020).

However, the discussion went on. Over the summer, one of Fauci’s key talking points was that any challenge study, should it be pursued, would have to go through rigorous scientific, ethical, and regulatory approval (Grady 2020). The second talking point was that challenge studies would only be used if there wasn’t enough virus circulating to complete the preferred randomized controlled trials (Grady 2020). This was echoed by other parties with NIH associations including an independent working group convened as part of Accelerating COVID-19 Therapeutic Interventions and Vaccines (ACTIV), a private-public partnership (Deming et al. 2020, Grady et al. 2020). The final talking point was that despite skepticism, the NIH was nonetheless cautiously making preparations in case challenge studies were needed. In mid-June, Fauci announced: ‘It’s on the table. I hope we won’t have to use it… We are making challenge doses. We’re not saying we’re going to use them’ (Owermohle 2020).

In July, Fauci asked Memoli to put together a team and begin writing a protocol for a potential challenge study (Memoli 2021a). Then in August, Facui announced that the NIH had awarded a contract to the BioMARC lab at Colorado State University to manufacture a GMP viral strain. However, he said a challenge model would only be used in an ‘absolutely far out contingency’ where the virus was not widely circulating (Gupta et al. 2020). He called challenge studies a ‘Plan C or D’ (Johnson 2020). BioMARC did go on to manufacture the strain for the NIH, and Memoli did write a protocol. However, there was never any real intention to use the protocol or the strain, and the virus wasn’t ready until after the authorization of the first vaccines (Memoli 2021b).

Officials at the NIH including Collins, Fauci, and Mascola, the director of the Vaccine Research Center, defended their caution about challenge studies with common justifications. A challenge model would take too long to develop. Challenge studies were not practically feasible and too risky to volunteers. There was no treatment or rescue therapy if someone did get seriously ill. Conventional randomized trials were moving quickly and they would produce better data anyway (Corey et al. 2020, Griffin 2020, Guarino and Johnson 2020, Grady 2020, Collins 2020). In the end, the conditions seemed to justify their risk averse position. In May, when Operation Warp Speed was announced, in July when phase three trials started, in September and October when case numbers were growing, and in November when phase three data revealed vaccine efficacy over 90%, challenge studies for FDA authorization proved unnecessary. External factors made the NIH’s decision for them. The first generation of safe, effective vaccines were developed and tested in 11 months. It would have been difficult for challenge studies to have meaningfully sped up the timeline to licensure or distribution.

Still, supporters of challenge studies questioned the NIH’s approach. They argued that it led to unnecessary deaths and could have led to many more if circumstances turned out differently (Morrison 2020b, Tabarrok 2020, Flanigan 2021, Yglesias 2021, Fraser-Urquhart 2021). They feared what could have happened had the worst case scenarios imagined in March and April come to pass. If the first vaccines failed, if they weren’t as effective as they turned out to be, if they weren’t as effective in some populations as others, if trials didn’t reach their endpoints as quickly as they did– supporters argued that in all of these situations people would have died when a challenge trial might have saved lives. Certainly things could have turned out differently. Supporters rightly argued that the world was lucky this time.

In the US, it seems that only the NIH had access to the facilities, the in-house expertise, the network of researchers and research sites, and the funding to undertake a project like this.^5^ Their reach operated on a second level too. More serious interest from the NIH might have eased concerns and brought not just pharmaceutical companies and vaccine developers, but philanthropic foundations, universities, and others into a collaboration, more like in the UK (Plotkin 2021, Rapeport 2021). The NIH brought credibility and legitimacy. It was a government agency, a respected research partner, and a trusted public institution. They could have mobilized their reputation to coordinate challenge studies and manage public confidence in them. However in the process, the NIH ran the risk of losing public confidence and losing public trust on a large scale. It certainly ran the risk of setting back challenge study research, but there was even greater fear that trust in vaccines, medical research, and even science itself was at stake as well (Durbin 2021, Memoli 2021a, Plotkin 2021. *Key criteria for the ethical acceptability of COVID-19 human challenge studies* 2020, Shah et al. 2020, Jamrozik and Selgelid 2020, Kahn et al. 2020, Bambery et al. 2016, Hope and McMillan 2004).

An adverse event in a Johnson and Johnson challenge study could have seriously damaged their reputation as well as public perceptions of medical research and vaccine research. They had to be sensitive to these concerns. However, the NIH, unlike Johnson and Johnson, Moderna, or Pfizer, was responsible for and to the people, to the citizens of the United States and even the world. They were driven by a mission ‘to improve the health of the Nation,’ not to make profit (Mission and Goals 2021). Perhaps that responsibility requires that they take fewer risks. Perhaps they have to be more careful with the public’s trust even if it seems to leave valuable options unused. This might be an ethical question or it might be one for sociologists and historians. It’s unknown what exactly Collins or Fauci were thinking in 2020 and 2021 as they were making these decisions about challenge studies, but clearly the benefit was not great enough or certain enough to offset their perception of the risks.

The factors that made the NIH hesitant about conducting challenge studies were also what made them uniquely qualified to run them. They were in a unique position to maintain public trust, maximize social value, and justify the risks that volunteers were willing to take on. If a model was set up at the NIH and its timing aligned with the pace of other research, they could have tested vaccines like Johnson and Johnson’s with the goal of speeding licensure and distribution to the public. Under the right conditions, this benefit could outweigh the risks. However, added social value that could make a challenge study worthwhile could also come from other places. Whether studying public health interventions like masking and transmission, studying fractional dosing and mixing vaccines, or prioritizing cheap, easily distributed, non-profit, or non-patented vaccines, there are numerous strategies beyond speeding first generation vaccines that might balance the risks of a challenge study with its unique benefits to society, as I’ll discuss below.^6^ It’s unlikely a pharmaceutical company would engage with these strategies because none are likely to benefit the bottom line. However, a truly independent institution working in the public interest and not in the interest of executive and shareholder profits might create a platform where this research and testing could take place.

In other words, because the NIH was a public institution whose mission was not financially driven, the uses and users of a challenge model there might have prioritized research that served people and science worldwide. By publishing findings openly, honestly, and transparently they could further contribute to the scientific field in myriad ways, some of them as yet unpredictable (Elliott 2020, Rid and Roestenberg 2020). This is not just a matter of individual values or intentions. It is not a matter of whether Fauci or Collins wanted to run challenge studies or not. It is a matter of the structural features inherent to public versus private, for profit institutions. An institution whose responsibility is to the people and not to shareholders, customers, or the bottom line might still use a challenge study to speed corporate vaccine development if that was going to generate the most social value and help the most people. However, it could also leverage its institutional structure and institutional identity in service of equity in vaccine development.

By the very nature of the institution and its responsibility to the public, the risks for the NIH were high, but the benefits were too. An NIH-sponsored challenge model, even if it couldn’t speed development of a first generation vaccine, could have generated unique social value that market-driven research–challenge studies or otherwise–might not. If the pieces fell into place, all of these uses could potentially have saved lives. Looking at institutional contexts, we can see the range of factors beyond sheer numbers of lives potentially saved that shaped and could have shaped their decision making and their construction of social value.

### The human challenge Programme

As in the US, individuals in the UK began thinking about challenge studies for Covid-19 as early as February of 2020. By May, Kate Bingham, the newly appointed chair of the government’s Vaccine Taskforce, had asked Garth Rapeport, an expert in respiratory disease with experience in challenge studies, to convene a group to explore the idea of running human challenge studies for Covid-19. Over the next year, Rapeport and the Taskforce brought together a range of partners including hVIVO, a contract research organization that specialized in challenge studies; academic researchers from Imperial College, University College-London, Southampton University, and Oxford University; and the Royal Free London NHS Foundation Trust to have these conversations and make the relevant preparations (Rapeport 2021).

On one level, the difference between the NIH and the UK efforts was that the UK government was in support of challenge studies, granting £33.6 million to fund research and preparations including viral production, reservation of facilities, and the dosing study conducted by Imperial College (Grover 2020). However, the belief that challenge studies had uses beyond testing the first vaccines and the broad collaboration among groups from various corners of the scientific world were as fundamental to the effort as the financial and political support from the government. Indeed, hVIVO and researchers at Imperial College worked at risk through the spring and summer without any certainty that challenge studies would garner government support, ethical approval, or ever take place. There was a belief that they should be pursued–that the virus should be manufactured, that facilities should be arranged, and that groundwork should be laid with regulators and ethics bodies–but there was also recognition that in the end maybe they wouldn’t and even shouldn’t actually be run (Rapeport 2021). Joining that chorus was Sir Patrick Vallance, one of the architects of the Vaccine Taskforce, who in July acknowledged that challenge studies were a valuable tool with a long history. He went on to suggest: ‘It is absolutely the right thing to explore, but we are not there yet in terms of having all the answers’ (Massey and Powell 2020).

Around the same time, the real risks of a challenge study were becoming better understood through research conducted by the National Health Service. The structure of the NHS enabled rapid, well-designed, and well-executed clinical trials and data collection on Covid patients that painted a clear picture of the risk to volunteers of infection with the disease. Based on data from the NHS and the International Severe Acute Respiratory and emerging Infection Consortium (ISARIC), researchers at Oxford developed a living risk prediction algorithm, known as QCOVID. The Imperial team used this algorithm in the early phases of their challenge model development and also used it in the informed consent process conducted with volunteers (Rapeport et al. 2021). The algorithm calculates one’s risk of hospitalization or death based on age, biological sex, ethnicity, BMI, and a range of risk factors (Clift et al. 2020). In the end, the team at Imperial set their inclusion threshold at that of a 30-year old with no risk factors who had a 1 in 250,000 chance of death and a 1 in 4,902 chance of hospitalization (Rapeport et al. 2021). Where the reported mortality rate in March was around 0.03%, by fall, the comprehensive NHS data made clear that it was far lower than that.

The approach taken by the researchers and administrators of the Vaccine Taskforce, Imperial College, and other partners was one that proceeded with caution, but it proceeded deliberately. For the UK researchers, as low as it was, the initial mortality estimate was not acceptable. Yet they did not take that as an intractable number or one that automatically made challenge studies obsolete. Instead, they acted on the belief that their understanding of the level of risk could change whether through better data, better treatments, or other methods of risk minimization including carefully refined subject selection. They also acted on the belief that the benefits of a challenge study were not restricted to testing the first generation of vaccines. They operated with few assumptions and moved steadily without rushing so as to get the details of everything from the strain to the protocol right. If, when the time came, the benefit outweighed the risk for all partners involved, then they would move forward. If not, they wouldn’t (Rapeport 2021, Rapeport et al. 2021).

That said, the benefits and the risks were constructed differently by different partners within the consortium. For one, hVIVO stood to benefit financially in ways that others didn’t. In shareholder meetings throughout 2020 and 2021, Cathal Friel, the CEO of hVIVO’s parent company, regularly highlighted how profitable Covid-19 challenge studies could be and seldom discussed either ethical questions or reputational risks. In part, this was because hVIVO simply faced fewer reputational risks. They even took pride in their willingness to take risks. In July, Friel explained to shareholders that hVIVO and SGS, a Belgian CRO, were the two world leaders in commercial challenge study research. However, he said it was ‘lucky for us. They don’t want to take the risk of messing around with Covid. We do, and they’ve left the market open to us’ (Proactive 2020). This tone may have been a product of Friel’s personality. However, a deeper institutional structure and institutional identity is evident in the discourse and decision making as well. hVIVO was not responsible to the public and didn’t risk public trust the same way the NIH did. Further, unlike either the NIH or a major pharmaceutical company, Friel projected the ethos of a startup that thrived on a climate of risk and took pride in its identity as a company made up of risk-taking entrepreneurs and innovators. Like Imperial College, hVIVO began preparing for challenge studies long before they could have known the investment would pay off, though they did so with a different assessment of benefits and risks. hVIVO is good evidence that the success of the UK effort was contingent on the convergence of many different partners with unique motivations, capabilities, and constructions of risk and benefit.

Similar to hVIVO, Kate Bingham of the Vaccine Taskforce brought an approach from outside of government or academic science. She came to the Taskforce with a background in life sciences and venture capital rather than medical research, public health, or science policy. Explaining her ‘VC mindset,’ she told the *Financial Times* that in finance: ‘we’re always dealing with risk and uncertainty. So we have incomplete data, and you have to make expert judgments. . And we do things very quickly’ (Cookson 2021). She was primarily discussing the UK government’s decision to spend hundreds of millions of pounds on the purchase of unproven vaccines, but it’s not hard to see how that logic applies to the challenge studies which she also championed (Rapeport 2021, Human Challenge 2020). It’s a very different mindset from that of the NIH which was led by Collins and Fauci, both academic scientists and career civil servants.

Nonetheless, upon announcement, the UK government treated these studies much like any other innovation in medical research. They didn’t immediately address or try to manage the perception that these studies were risky, controversial, or the subject of ethical debate. In a press release from October 20, 2020, Alok Sharma, the Secretary of State for Business, Energy and Industrial Strategy, said:

We are doing everything we can to fight coronavirus, including backing our best and brightest scientists and researchers in their hunt for a safe and effective vaccine. The funding announced today for these ground-breaking but carefully controlled studies marks an important next step in building on our understanding of the virus and accelerating the development of our most promising vaccines. (O’Hare 2020)

Here and elsewhere, the UK government expressed pride in the initiative, in the scientists leading it, and in the contribution to medicine they were going to make for this pandemic and beyond (Department for Business, Energy & Industrial Strategy 2020). Like the government, Chris Chiu, the principal investigator at Imperial College, and Andrew Catchpole, the chief scientific officer at hVIVO, favored a discourse of innovation and preparedness over one that addressed risk and ethical controversy (O’Hare 2020, Magee 2020). They didn’t focus on the altruism of volunteers, nor did they make promises about the lives that would be saved through a challenge study. Throughout the course of the initial study, they focused on science and safety (Killingley et al. 2022).

However, there was still skepticism about the studies from other corners. Seema Shah, Megan Deming, and Francis Collins were among those who were uncertain that what scientists would learn from running challenge studies in 2021 would be worth the risk involved (Callaway 2020, Booth and Johnson 2020). John Moore, a professor of microbiology and immunology at Weill Cornell Medical College (himself British), was also skeptical of what a lab-based challenge model could reveal about the virus as it circulates naturally. In a *New York Times* article, he went on to argue that it was politics advancing this dubious approach: ‘There’s unquestionably vaccine nationalism involved… It’s a race for money and glory. That’s the reality of it’ (Mueller 2020). In a broader discussion about challenge study stakeholders, Shaun Griffin and Hugh Whittall of the UK Pandemic Ethics Accelerator also asked about the role of ‘politicians who are keen to ensure the UK is seen as the global frontrunner in conducting pandemic research’ in decision making (Griffin and Whittall 2021). The UK government’s public comments may have emphasized science and safety, but their actions were easily read through the context of British politics and the eagerness to capitalize on the success of the homegrown Oxford/AstraZeneca vaccine for nationalist, as well as scientific, ends (Haseltine 2020, Behr 2021, Department for Business, Energy & Industrial Strategy 2020).

Ultimately, the decision to run or not run Covid-19 challenge studies in the UK or anywhere else was more than a matter of the research being ethical or unethical, though of course that debate was central (Davies 2021). On the scientific side, there was clearly a stronger belief among the UK researchers and stakeholders that challenge studies were not just a tool for testing a first generation vaccine, as was often the perception in the United States. On the ethics side, what the UK example shows even more vividly than the others is that when ethical considerations are as complicated and contested as this, decision making is contingent on a range of social, institutional, and political contexts rather than on the obvious risks, benefits, and ethical principles alone. The initiation of the UK effort was not just the product of collaborating parties agreeing on the nature of the value of a challenge study. It was also the product of complicated and contested factors coming together in everyone’s interest. Social value was constructed differently, but more to the point, value wasn’t just social. It was also financial for hVIVO. It was also political for the UK government. Just like the decisions made by Johnson and Johnson and the NIH, those made in the UK were shaped by the interests, identities, and responsibilities, as well as the risks and benefits, of relevant parties whether governmental, commercial, or academic institutions.

## Secondary uses

If the risks and benefits of a Covid-19 challenge study were constructed differently across institutions, they did all agree that in order to proceed, a challenge study had to have social value, and it would have social value if it could save lives. The emphasis placed on lives saved made it a narrow definition of a concept that often referred broadly to the value of improving health. Nonetheless, it reflected ideas that dated as far back as the Nuremberg Code’s affirmation that “experiments should be such as to yield fruitful results for the good of society” (Nuremberg Code 1996, Council for International Organizations of Medical Sciences 2016, Emanuel et al. 2000, Rid and Shah 2017). There is a relevant distinction to be made here. In research, there are benefits (and risks) which accrue to an individual or, as we’ve seen, to an individual institution. These are relatively easy to describe once they are identified. Then there is social value, a concept which invokes a broader contribution to society and to humanity, and thus is more difficult to measure (Rid 2020). Indeed its vagueness was part of its discursive power during these debates. That said, for Johnson and Johson and the National Institutes of Health, the source of the social value of a potential Covid-19 challenge study was clear. It was rooted in the life saving benefit of testing one of the four frontrunner vaccines.

However, just as with risks and benefits, there was more than one way to construct social value. None of these were mutually exclusive, but different efforts sometimes had different emphases. The British consortium had a more expansive understanding of the potential value of a Covid-19 challenge study. They highlighted the benefit to society of expanding scientific knowledge which could, eventually, improve health and save lives even if it did not do so immediately.

The social value of a Covid-19 challenge study could also be constructed in terms of vaccine equity. In a more general context, scholars have argued that because social value is a matter of both benefits and beneficiaries, the people who are worst off–usually those in the developing world–should be prioritized in order to maximize that value (Barsdorf and Millum 2017, Rid and Roestenberg 2020). In a related discussion, Rahul Nayak and Seema Shah argue for a conception of global social value that emphasizes the duties of global beneficence and distributive justice which obligate research institutions–at least publicly funded ones–to support research that will benefit people worldwide, not just those in wealthy nations or those where the research takes place (2017). Some advocates of Covid-19 challenge studies adopted a version of this approach, framing challenge studies as research with just this kind of global social value (Dias et al. 2020, Morrison 2020a, Savulescu and Rohrig 2021, Impact 2022). The universal understanding was that a challenge study that could test a vaccine faster would have immense social value. The hope, expressed by potential trial volunteers, was that it would speed vaccinations for everyone, everywhere and benefit all of humanity including people in the developing world (Belton 2020, Gokey 2020, Roex 2020). The social value of a challenge study was high because the number of beneficiaries was high and because so many of those beneficiaries might come from among the worst off. Of course, realizing that promise would have been difficult. Even if a challenge study could in fact have sped vaccine development by a month or more, the reality of the existing regime of global inequality is such that even if lives could have been saved through a challenge study, distribution would not have prioritized the worst off as many would have hoped. Realistically, social value would have remained limited on those grounds (Evans 2020).

Still, there were other uses for challenge studies which could have had unique benefits and generated global social value that prioritized the worst off. The selection of which vaccine would be tested was one. Among the problems with the Moderna and Pfizer vaccines was that they were expensive, had restrictive cold chain requirements, were complex to manufacture, and/or had rigid intellectual property enforcement. All of this limited the number of people they were likely to reach and thus limited their social value globally despite high levels of efficacy. Other vaccines could potentially have shifted the ethical calculus of a challenge study and shaped vaccine manufacturing and distribution by addressing the limitations of the mRNA vaccines developed by pharmaceutical companies. Those who were selecting which candidates would be tested in the challenge model could have prioritized the development and testing of low-cost vaccines, one shot vaccines, or vaccines with no cold chain requirements, all of which could have been easier to make or distribute in the developing world. If it was a public institution making these decisions, they might even be obligated to set priorities this way.

A sponsoring institution could also have prioritized vaccines from university researchers or non-profit institutions over pharmaceutical companies. These groups didn’t have the deep pockets of a rich corporate entity or the resources of Operation Warp Speed. Thus, a cheaper, faster approach to early phase testing could have been helpful in providing proof of concept for further investment. This is a key use for challenge studies outside of the pandemic context as well. Further, prioritizing candidates from these kinds of institutions could have meant prioritizing developers that pledged not to profit off their vaccine or chose not to patent their vaccine altogether. In addition, if these were vaccines that were easily manufactured in existing facilities around the world, developing countries could take extra advantage of the lower barriers of intellectual property to produce their own supply. A challenge study that aided in vaccine selection could theoretically shape the system in which vaccines are funded, tested, and manufactured even if it had little influence on allocation. Eliminating intellectual property and prioritizing vaccine recipes that could be produced locally in the developing world could have had the potential to shift power away from corporations and Western gate keepers of distribution. In reality, however, there was no inclination toward such priority setting on the part of the NIH, Operation Warp Speed and its vaccine developers, or the UK Vaccine Taskforce.

Finally, as many pointed out, a challenge model could also have been used for research outside of the initial phases of vaccine development and testing to ask questions whose answers could benefit everyone, no matter where they were located. Testing fractional dosing to allow the same volume of vaccine to vaccinate more people was one possible use. This could certainly benefit the global public more than a private corporation. Studying transmission, immunology, and other aspects of the disease could garner knowledge that might aid clinical treatment, drug development, and public health policy around the country and around the world. Indeed, the first findings of the Human Challenge Programme included observations about exposure and masking (Killingley et al. 2022). Further, researchers and administrators could have developed the model and the challenge strain and made them available to researchers at no cost so they might answer their own questions. They could have also chosen to make data and protocols open and available, again allowing researchers to answer their own questions, rather than focusing on the goals of a pharmaceutical company testing its product.

Neither Johnson and Johsnon nor the NIH constructed the benefits and social value of Covid-19 challenge studies on these terms. Neither was interested in using a challenge study for anything other than the faster authorization of the first generation of vaccines which were already pre-selected by Operation Warp Speed. They constructed social value along these lines in accordance with their views of the science and ethics as well as their specific institutional interests and concerns. Because of this, these secondary advantages which could encourage the construction of social value on a global scale were not enough to shape their calculations despite the responsibility of the NIH–and Johnson and Johnson as a company that accepted government funding from Operation Warp Speed–to the American people and the world wide community. The benefits of these approaches seem to me to be too hypothetical and too contingent on other factors to say any institution–public or not–was obligated to conduct Covid-19 challenge studies. However, it’s worth wondering how different the debate might have been and how different vaccine development could have been if institutions had been more inclined to this way of thinking and this construction of social value.

A challenge study would not have been a panacea, and it certainly would not have been a solution to global vaccine inequity. Even if a challenge study could have been a useful tool during the acute phase of the pandemic, these uses are limited workarounds to unequal relations of power. The risk of such research could be unnecessary in a different regime. Further, those systems operate on a scale so large and so wide that, even in the best case scenario, a study like this might have limited impact. None of these benefits and none of the social value that could accrue from a focus on equity would necessarily justify a challenge study on their own. Further, the consideration of these factors says nothing about the magnitude of risk or the autonomy of the volunteer and how those principles should be weighed in decision making. However, when considering the benefits and social value of a challenge study for Covid-19 or any future pandemic disease, it’s valuable to paint an expansive picture of its potential and what it is that a challenge study can, at least in theory, uniquely accomplish for science as well as for people around the world. Just as we should analyze the ways in which these institutions did construct risk, benefit, and social value, we should also analyze the ways in which they did not.

## Conclusions

With any challenge study, including those for Covid-19, decision makers make two decisions. They decide whether to create and use a challenge model, and they decide what to use it for. For Johnson and Johnson as for the NIH, the only use that was potentially worth the risk was accelerating development of a first generation vaccine from one of the existing frontrunners. When it became clear that traditional phase three trials were going to be the best and fastest option, neither saw any reason to pursue the development of a challenge model further. Maybe they were right to do so. A challenge model wasn’t needed for the first vaccines, and whether there was any scenario in which it could have helped with their authorization is debatable. The value of other uses was equally uncertain. Even a non-profit, low cost, easily manufactured, patent-free vaccine that did show promising results in a challenge study would probably still need phase three testing that could be costly and time consuming.

The NIH was the only institution in the United States that had the resources, the infrastructure, and the public identity to run a challenge study that might realize the full potential of this tool. However, throughout the pandemic, both the NIH and Operation Warp Speed failed to use their leverage to support global vaccine equity. It’s then hard to imagine they would use a challenge study for that purpose. In the UK, Kate Bingham pointed to investment in vaccines that were cheap and easy to make and distribute as a key priority for future pandemic responses (Bingham 2020). Though she didn’t connect that explicitly to challenge studies and she has since left the Vaccine Taskforce, it remains to be seen if her suggestion has any bearing on what subsequent iterations of the Taskforce do with the three slots they’ve reserved in the Imperial model at the Royal Free Hospital.

Throughout this essay, I have used critical theory and approaches from science and technology studies to tease out the social, cultural, political, and economic contexts in which assessments and constructions of risk, benefit, and social value were made with regards to Covid-19 challenge studies. The approach may or may not be useful in making ethical recommendations or ethical judgments strictly speaking. However, it does provide important context for thinking about challenge studies, medical research, and pandemic decision making in terms of the larger systems and institutional structures in which they operate. Pulling back in this way reinforces the importance of examining both research subjects and research sponsors. Sponsors are not individuals but institutions. Decision making is thus shaped by the rules, logic, and discourse embedded in larger systems of knowledge production, priority setting, and responsibility to society. I hope my incorporation of social history and science and technology studies alongside bioethics is a productive addition to growing bodies of literature on risk, benefit, and social value in challenge studies, medical research, and the Covid-19 pandemic.

## Electronic supplementary material

Below is the link to the electronic supplementary material.

Supplementary Material 1

Supplementary Material 2

## Footnotes

1 However, as Ben Bramble and George S Heriot and Euzebiusz Jamrozik point out, this raised its own ethical questions (Bramble 2021, Heriot and Jamrozik 2020).

2 Their position was also unique because of past experience. They’d had success with a respiratory syncytial virus challenge study in 2018, making them more comfortable with the approach. They also feared a repeat of their Ebola vaccine testing which failed to reach its endpoints due to the waning of the outbreak in West Africa in 2015 (Morrison 2021a).

3 A partial exception was Janssen’s interest in using a challenge model to establish correlates of protection that could have been of wider service to researchers and vaccine developers if the data was shared publicly (Morrison 2021c). While there is reason to be skeptical that they would actually share data publicly, Johnson and Johnson did make a unique commitment to practicing open science in 2014 which gives reason to be a bit more optimistic about their potential actions (Herper 2014).

4 The larger story of Johnson and Johnson’s vaccine is a bit more complex than this. Their decision to focus on a one-shot vaccine without extreme cold chain requirements can certainly be read as a nod to global health (Zimmer 2020). In addition, they did pledge to sell their vaccine on a not-for-profit basis, but only for “emergency pandemic use” indicating that that pricing could end at their discretion (Johnson and Johnson 2021b). Ultimately Johnson and Johnson failed to turn the profit or have the impact they hoped for because of bad PR around adverse events and lower efficacy than the mRNA vaccines (Herper 2021).

5 The NIH effort can be compared to that of 1Day Sooner, a non-profit organization whose mission was advocating for Covid-19 challenge study volunteers. They also attempted to manufacture a virus, but did not succeed in part for these reasons.

6 A challenge study could also aid in the development of universal coronavirus vaccines. Despite the hesitance in 2020 and 2021, researchers and administrators at the National Institutes of Health, including Anthony Fauci, suggested this as a future, post-pandemic possibility (Morens et al. 2022).
