# Supplementary material for: Risk, benefit, and social value in Covid-19 human challenge studies: pandemic decision making in historical context
Source: Monash Bioeth Rev. 2022 Jun 15;40(2):188–213. doi: 10.1007/s40592-022-00156-6 (PMC9200217; doi:10.1007/s40592-022-00156-6)
Supplement: Supplementary file 1 — Supplementary Material 1 [file 40592_2022_156_MOESM1_ESM.docx]

Mabel Rosenheck

[Rosenheck@gmail.com](mailto:Rosenheck@gmail.com)

Justin Oakley, Chief Editor

Euzebiusz Jamrozik, Assistant Editor

Monash Bioethics Review

March 18, 2022

Dear Editorial Team (Attn: Zeb Jamrozik),

Thank you for your consideration of the revisions for the article “Risk, Benefit, and Social Value in Covid-19 Human Challenge Studies: Pandemic Decision Making in Historical Context.”

I am the sole author and this manuscript is my original work. I confirm that this manuscript has not been published elsewhere and is not under consideration by another journal. I have approved the manuscript and agree with its submission to the *Monash Bioethics Review*.

Regards,

Mabel Rosenheck, PhD
