# Supplementary material for: Risk, benefit, and social value in Covid-19 human challenge studies: pandemic decision making in historical context
Source: Monash Bioeth Rev. 2022 Jun 15;40(2):188–213. doi: 10.1007/s40592-022-00156-6 (PMC9200217; doi:10.1007/s40592-022-00156-6)
Supplement: Supplementary file 2 — Supplementary Material 2 [file 40592_2022_156_MOESM2_ESM.docx]

Mabel Rosenheck

Responses to Reviewer Comments

“Risk, Benefit, and Social Value in Covid-19 Human Challenge Studies: Pandemic Decision Making in Historical Context”

## Reviewer #1

Reviewer #1: Thanks for the opportunity to review this manuscript. Overall, I think it will be an important contribution to the literature. I enjoyed reading it. It provides an important overview and analytical synopsis of where we are with challenge studies in present day. I've given it 'major' revisions as a recommendation but I actually think it's on the border of minor/major, for what that's worth. I hope these comments are useful to the author.

Major comments:

1. Although the author notes at the outset and end that they're approaching the topic of challenge studies from a critical theory approach, not a normative ethics/Western analytic tradition, the conclusion section is highly prescriptive of what we ought to do regarding considerations of justice and challenge studies as it relates to the idea of social value. That's generally fine but there are a few issues that arise: first, the type of argument changes drastically in the last quarter or third of the paper relative to the descriptive analysis done in the first third or first three-quarters. I wondering if there's a better way to set this up, stylistically speaking. But second, and more importantly, the arguments could be more robust if they author told us what notion of justice they're adhering to. The normative arguments in the conclusion section lacks the rigour of the proceeding parts. So we - the readers - don't know what the author means by justice, or what this means in application (same with other concepts like trust, for example). For example, the author states on page 27, lines22-27 "the use of a challenge model could increase its social value by centring justice as well as raw utilitarianism and maximisation of benefits". But it's not clear what the author means by utilitarianism given the various permutations of utilitarianism that exists, making that claim difficult or impossible to evaluate. A third issue with this conclusion section is that it doesn't speak to the previous case studies in a direct manner. It would have strengthen the paper to tie the conclusion and arguments about justice more explicitly with the three case studies

2. Following on from the previous point, key terms are rarely defined or described, particularly the idea of social value. Again, I suspect that's in part intentional since much of the paper is about how various entities understood the concept of social value in the context of COVID and challenge studies. That's fine, but I still think it would strengthen the paper for the author to tell us how social value is generally understood in research and research ethics, e.g., CIOMS Guidelines or by various authors throughout the decades. Moreover, doing so would be important since the author uses the notion of social value, still undefined, to make normative arguments in the conclusion, which leads to ambiguity, as discussed in the previous point.

**I revised the section titled “Secondary Uses” and incorporated most of these remarks. The section now includes a definition of social value with relevant references. I also draw a distinction between the risks and benefits which I focus on in the examples and this section which explores social value as a related, but separate concept which also has been constructed by the different parties in different ways. (I mention this structure in the introduction as well.) I identify those constructions by J&J/NIH, the UK, and supporters (especially volunteers) who advocated using challenge studies as a tool of vaccine equity. I go on to highlight the uses that can facilitate equity as a way of constructing social value.**

**I stay more general in a discussion of equity rather than narrowing in on specific conceptions of justice.**

Minor comments:

1. pg 5, line 9: "They also saw that if the solution was going to be vaccines" not sure how many people thought/think that vaccines alone would be *the* solution. An important part, no doubt, but might consider rewording.

**Reworded.**

2. Bottom of page 13, who's the 'they' in the third sentence: J&J or scientists/ethicists/administrators?

**Clarified**

3. pg 20, line 13 "the level of risk could change whether through better data..." The level of risk wouldn't change but our understanding of the risk might change through better data. Might want to reword, I think.

**Reworded.**

4. Top of page 22: Not sure the extent to which the block quote on page 21 or the quote by Catchpole actually support the conclusion the UK gov wasn't focusing on altruism or lives saved but rather science and safety. The passage in the block quote specifically could really be read to suggest they were very concerned with social good in the sense of lives saved, though not explicit.

**Yes, they definitely were presenting the studies as being valuable in the sense that they could save lives. The emphasis here is that that discourse of the government and those involved at Imperial and hVIVO did not address the controversy of the ethical debate head on. They did not address the specific terms or the arguments from critics, understandably. They didn’t actively reassure people that these volunteers were not being exploited and this study would balance risk and benefit appropriately. In general, they didn’t highlight the risks.**

5. Language choice: there are passages throughout the paper where the language is a bit flowery for my taste. I leave this for the author to discuss with the editors, but for example the passage on page 22 lines 51-53 read a bit trite. (Maybe I need to loosen up, could very well be the case.)

**Revised.**

## Reviewer #2

Reviewer #2: Thank you for the opportunity to review this engaging manuscript focussing on the construction and consideration of concepts of risk, benefit, and social value in relation to COVID-19 human challenge studies.

The central point is well made, that risks, benefits, and value are construed differently depending on stakeholders' perspectives and shifting interests. I appreciated the simple statement that challenge trial decision making comes down to 'whether to create and use a challenge model, and…what to use it for,' and, framed slightly differently earlier in the piece, whether a model is safe enough and useful enough.

Overall, the manuscript was enjoyable and engaging to read, and the analysis and discussion was strong. I don't think the thoughts I offer below include any absolute requirements to make changes to the manuscript, or even to respond to each and every comment, but rather are a series of ideas and questions that may possibly help the author to improve a manuscript which is already good. Apologies for the repetition.

My concerns and comments mostly relate to the occasions where the manuscript veers towards the unfortunate popular characterisation of challenge studies as life and death, all or nothing, perfect or without any value at all. Similar false dichotomies have affected virtually every intervention discussed during the pandemic. There are also some important unstated assumptions that permeate the piece, of relevance for future considerations. Perhaps most importantly, echoing the discourse through 2020 in particular, is the (false) comparison of human challenge trials done according to conventional timelines with 'Warp Speed' development of vaccines without challenge trials. In considering the place of the human challenge trial platform in the future of pandemic preparedness, critical questions are: what might warp speed human challenge trials look like, and can they be integrated into the platform, prototype and target pathogen paradigm so that the discussion and development of models does not have to start from scratch in the intense initial months of a future pandemic?

**I tried to address these concerns by making clear that the perfect/without any value distinction was one made by institutions like J&J and the NIH (and by media discourse), not one that is reflective of the role challenge studies could actually play. I also clarified throughout that the UK consortium was more in line with the reviewer’s thinking.**

**I moved away from the future considerations as I felt I had less to contribute there and those questions are better left to a different kind of expert.**

(Consider if, like the vaccine platforms, there had been a standing coronavirus challenge platform capacity to allow almost immediate challenge trials testing the first vaccines with promising phase 1 safety using a GMO common cold beta-coronavirus with the SARS-CoV-2 spike protein… with use of SARS-CoV-2 itself once acceptable risk calculations were possible)

Challenge research belongs in our broader concepts of clinical trials for vaccine development, and the ethics of challenge trials should not be separated from other early phase research. Beyond the 'yuck factor', there are very few differences. Arguably, by March 2020 we already knew more about this novel coronavirus than for any experimental immunomodulatory monoclonal antibody tested in first-in-human phase 0/1 trials, yet those are not controversial (except when there are spectacular catastrophic outcomes - which have NEVER been seen in a human challenge trial).

**These points are well taken, but I felt they were beyond the scope of the article.**

ABSTRACT

"The risks of deliberately infecting healthy volunteers with a deadly pathogen were not low, but the benefits could have been immense."

Many, perhaps most, human challenge trials study pathogens associated with severe and fatal syndromes. Without the nuance included later the 'deadly pathogen' characterisation here is emotive and unnecessary, I think. In truth, the risk to healthy volunteers, as discussed later and borne out in the early results from the UK trial, were low. Perhaps including an introductory qualifier such as, 'In the broadest sense and across all stakeholder groups, the risks of deliberately infecting healthy volunteers with SARS-CoV-2 were not low, but the potential benefits could have been immense.' Or something like that?

**Point well taken, but I felt it was unnecessary to address this in the abstract. It may be emotive, but I think that’s the point.**

In the sentence listing J&J, NIH, UK government, I would recommend identifying each even more clearly, 'Preparations were separately undertaken by the pharmaceutical company Johnson and Johnson, the United States National Institutes of Health, and a British public-private partnership.'

**Incorporated.**

On first reading, the term 'risky research' made me uncomfortable, although I understand it has an understood meaning and has been used and discussed previously in relation to this pandemic. All context considered, COVID-19 human challenge trials were more or less 'risky' for different stakeholders and for different reasons, as I think this manuscript nicely illustrates. But on conventional clinical trial ethical lines considering participant benefits, harms, and autonomy, I'm unconvinced that challenge trials are an intrinsically riskier than other clinical trials. Certainly, the most dramatic early phase safety catastrophes have been in phase 1 drug trials, not human challenge trials. An additional reference to 'risky research' or further discussion somewhere later in the manuscript may be appropriate? Perhaps the piece by Chappelle and Singer in Research Ethics? (https://doi.org/10.1177/1747016120931920).

**I revised the language in the abstract, but in general, while I think these points are well taken, I felt they were beyond the scope of the article. I did, however, clarify in throughout that the risk was to the volunteer.**

INTRODUCTION

Lines 13/14 - infectious diseases are always considered in terms of the relative contributions of pathogen, host, and environment/setting. I would include some mention of the pathogen dynamics in this line, and rather than 'vaccines and treatments' I'd suggest 'preventive and therapeutic interventions'. Because… challenge trials can answer questions about pharmacological and non-pharmacological interventions.

**Incorporated.**

Line 20/21 - I wouldn't say that researchers have used human challenge studies for centuries. Rather, experimental human infection has been used for centuries. Why this distinction? Because it is a trap to consider modern human challenge studies as separate from other modern clinical research studies - we 'challenge' humans routinely with drugs, devices, vaccines, and other interventions.

**Revised to specify that they are a tool.**

Line 25-30 - The Hippocratic Oath primum non nocere reference here is the most literal of possible interpretations, of next to no relevance for modern clinical practice or clinical research. More often than not, we do do harm. That's an old discourse, isn't it? The idea of risk (or harm) parity in research is hardly a new one but human challenge trials aren't a form of ritual human sacrifice to Gods of utilitarianism ('safe enough? useful enough?' framing as above). In Matt Memoli's quote, should you indicate in square brackets who are 'them'? What Matt's formulation is missing is that the volunteers are there for the same reason - 'for the benefit of society and the world to generate data that can male everyone better in the long run'. In the Singer piece mentioned above, and virtually all of 1DaySooner's output, this point has been made very strongly - volunteers are stakeholders with a particularly valuable contribution to make regarding what are acceptable risks for participants.

**Point well taken, but more than I could tackle here. Described it as a “simplistic principle”**

**In general, the argument about volunteers as stakeholders is relevant, but not the focus of my discussion.**

P3, line 4/5 - it is an emotive rhetorical question I know, but important to underline that obviously nobody proposing human challenge trials sought to justify replicating the horrific global impact of COVID-19 in a SARS-CoV-2 challenge trial.

**Revised.**

There's no doubt there was an explosion of literature regarding the ethics of COVID-19 challenge studies, but was it truly 'rich'? A sizable portion of the commentary seemed to stem from a lack of familiarity with human challenge research, including from many ethicists. In most cases, the risks and benefits were overstated, flattened and simplified for effect.

**Revised to say “large” rather than “rich.”**

LITERATURE ON COVID-19 CHALLENGE STUDIES

P4, line 44/45 - any particular reason Matt and Meta are mentioned, but nobody in the UK? ('led by…')

**Added Garth Rapeport.**

P4, line 50 - worth including at least one reference for the 'growth and mainstreaming' of human challenge studies? (perhaps Meta's Lancet ID review?)

**Included.**

P5, line 60 - may be worth explaining 'under control' here… a good reference might be this https://pubmed.ncbi.nlm.nih.gov/33550606/ The point is that the early successes of public health interventions in preventing uncontrolled transmission in many countries made them unsuitable for phase 3 trials. Even in countries that completely failed to control transmission of the virus (USA, Brazil, UK), phase 3 trials were only possible with huge numbers of participants and still managed to only capture relatively few events in the key time period prior to emergency authorisation of the first vaccines.

**I address this elsewhere, especially the bottom of page 5.**

This begins to get at my key point that the 'Warp Speed' mindset (and accompanying investment) was decisive in accelerating development of COVID-19 vaccines. It is unknowable what would have been the impact of a similar mindset (and investment) applied to challenge trials.

P6, lines 33/34 - dexamethasone and remdesivir should not be capitalised. In any case, 'antiviral and anti-inflammatory therapies' is probably better here. After all, early antiviral monoclonal antibodies were ultimately most prominent in UK challenge study protocols (none were required).

**Incorporated.**

P6, line 38 - I'd remove 'rescue therapies' here. It's a loaded term of uncertain meaning, and certainly not an absolute requirement for challenge studies of many other pathogens. The point here is that, for a variety of reasons, the already low risk of serious harm to participants became even lower.

**I put this in quotes. I wanted to include it because it was an extremely common term in the discussion. It does deserve more attention than I can give it here.**

P6 - line 40 - the 'long COVID' coverage here is superficial, emotive, and treats disputed science as fact. There is very limited evidence 'that the lasting effects of even mild cases of Covid-19 could be truly devastating'. A better point would simply be that uncertain and disputed risks of long term impacts gave credence to arguments that doctors simply didn't…

**I adjusted the emotive language. I’m not a physician, but it doesn't seem to me that this is disputed science.**

P6 line 51 to P7 line 21 - all of these concerns apply to conventional preclinical, phase 13 studies as well. The difference is that the 'Warp Speed' mindset and investment completely blew away the cobwebs of 'non-pandemic levels of urgency, speed, and collaboration' for the conventional pathway. Eyal et al have previously done a nice job of comparing generalisability concerns between challenge and conventional vaccine trials.

I appreciate that this broader framing was not the intent of this manuscript, however it is really central to considering human challenge trials. They were put in a different box. Ethical concerns for challenge trials applied to conventional phase 1-3 trials too - they were largely shelved in pursuit of the first COVID-19 vaccines through more familiar clinical trials. Imagine a not implausible counterfactual in which the first 4 mRNA (Pfizer, Moderna) and adenovirus-vectored (J&J, AstraZeneca) candidates had failed in n=30,000 phase 3 trials and TTS and myocarditis signals had also been apparent. The entire construction of risk, benefit, and social value of challenge trials was separated from consideration of risk, benefit, and social value of conventional trials because of a broad misunderstanding that they are totally different - I'd speculate that the idea of deliberate infection triggers something deep, and everything that follows mostly stems from that primitive response.

**I did address the counterfactual on the bottom of page 9 and the top of page 10.**

Line 56, page 7 - the mRNA and adenovirus-vector vaccine platforms were not truly 'new', they resulted from a determined approach to pandemic preparedness. It would be more accurate and less informal to say, 'Successful deployment of flexible vaccine platforms, Operation Warp Speed in the US, massive advance purchasing agreements globally, and failure to control viral transmission in countries such as the US, UK, and Brazil…'

**Revised.**

EFFORTS

P8, line 5-18: It's accurate to describe the early discussion in these dramatic terms but any serious human challenge researcher would dispute that 'It was a real life trolley problem…' That is a completely inaccurate analogy of the risks entailed in a COVID-19 human challenge trial and the risks considered acceptable. It was 'a chance worth taking' precisely because it was almost immediately evident that participation was not a suicide mission.

**Revised.**

P9 - Again, Operation Warp Speed and advance purchasing agreements worldwide were calculated gambles made in the absence of efficacy data - downselection and manufacturing investment decisions were made without the human efficacy data that challenge trials may have provided. Those primary advantages of a challenge study weren't actually largely irrelevant until the emergency authorisation of vaccines made on the basis of efficacy data (they work) enabled administration of the doses manufactured early (they work, and we have them ready to give). I don't argue with the framing, just what the underlying assumptions mean for considering future uses of challenge studies. Again, consider the counterfactual where the first vaccines were not successful, despite the immense commitment to accelerated development represented by OWS and advance purchasing agreements.

**Addressed the counterfactual. I decided the future considerations were beyond the scope of the article. Hopefully someone else can draw them out.**

P9 - lines 46-49 - same point really - The phase 3 trials hadn't ended when their early efficacy results contributed to emergency authorization. Who really knows what all of that commitment and coordination could have done if applied to challenge studies? Under similar conditions and similar commitments to acceleration, following accelerated development of the model, a primary efficacy signal from a challenge trial could be available in 14 days. Again, the case for conventional trials was absolutely reliant on uncontrolled viral transmission in US, UK, Brazil - an ethical aspect that has been rarely considered. In any case, the challenge vs phase 3 argument has almost always been a false dichotomy. It is true that, not without risk, massive commitments essentially leapfrogged challenge trials for accelerating development of the first vaccines.

**“It is true that, not without risk, massive commitments essentially leapfrogged challenge trials for accelerating development of the first vaccines.” That’s really where my focus is rather than the question of how fast a challenge model could, theoretically, have been set up.**

The discussion through the Johnson & Johnson/Janssen section makes it even clearer - the motivations for pursuing challenge trials dissolved for private companies operating under the cover of wartime-levels of insurance provided by public investment. The same can be said for so many aspects, e.g. despite considerable uncertainty about the optimal dose and schedule for COVID-19 vaccines (for efficacy and safety), the schedule studies have been pursued almost entirely as phase 4 studies by public institutions and there is only extremely limited dose-finding data despite some potential dose-related adverse effects (e.g. myocarditis in adolescents). Underneath the veneer of apparent spectacular market success is this reality of market failure - private companies only delivered these vaccines because of massive public investment, and have not been bound by that investment to continue to undertake research in the public interest. Overall, I really enjoyed this section, with the focus towards the end on the profit and public relations motives of private pharmaceutical companies.

NIH section

P14

The first sentence harks back to the point that I've made - we license big pharma to make vaccines and make vaccine making decisions, and Western governments mostly adhere to laissez-faire principles when it comes to their interactions with big pharma, even, almost unbelievably, when the development pathway is underwritten by massive public investment. One might argue there's been a corporate takeover of the construction of social value…

P14, line 56 - again 'riskier' seems to refer to the mutable concepts at the core of the discussion here. Would 'unfamiliar' or 'unconventional' be more appropriate? Again, I'd suggest that our consideration of risk here would be wildly different if >150,000 volunteers had participated in negative phase 3 trials.

**Incorporated.**

P15 - Such an interesting insight into the reactionary and conservative mindset of the NIH. The 'absolutely far out contingency' and 'Plan C or D' quotes are great. At the same time as millions of Americans were participating in an uncontrolled human infection study on the streets, with disastrous consequences, the concept of a controlled human infection study of fewer than 100 informed healthy adult volunteers was simply too much to bear for NIH. They could comprehend and imagine a dramatically expedited phase 1 to 3 pathway but could not imagine expedited challenge model development.

P17 - other types of trials/uses that NIH might have used for a challenge model of limited interest to pharmas besides those listed: heterologous immunisation with different vaccine products; 'prime-pull' strategies employing parenteral and mucosal vaccine products that arguably could have prevented more transmission than purely parenteral products; testing the efficacy of masks/PPE without the oddity of mannequin studies, circumstantial smoke studies, or econometric statistical gymnastics such as the Bangladesh community cluster randomised trial; considering the relative contribution of different routes of transmission; informing public health interventions by truly understanding periods of incubation and infectivity. Many of these questions remain open after two years of the pandemic, and are reheated for every new variant. Of even greater interest would be challenge trials to accelerate development of pan-coronavirus vaccines (as has been done for pan-influenza vaccines) - a true 'moonshot' that the current COVID-19 vaccine developers have limited interest in pursuing.

UK programme

P19 - the foundational point made here is that there was a broad commitment in the UK to pursuing these studies, and not simply as a last resort 'hail Mary' contingency.

I think this section begins to make many of the points I've made above - that challenge trials can and should be considered alongside rather than separate from other clinical research.

P22/23 - science so often finds itself under-resourced, on the wrong side of assorted political motivations, that there is a real risk of overdoing this discussion of the UK government's motivations for supporting COVID-19 research, including challenge studies. Both Donald Trump and Jair Bolsonaro sought to garner political capital from COVID-19 vaccine development. As the author is trying to illustrate, these matters are always political, in part. It seems a long bow to draw to argue that this was decisive in the UK challenge initiative. Certainly in the scheme of COVID-19 related investments, the challenge program is a small one.

**Agreed. Revised.**

Conclusions

The justice/equity points made here feel like additional discussion rather than conclusions, not that I was particularly bothered.

More bothersome to me was the repeated framing of challenge trials as some kind of last resort.

The point is well made that challenge trials are helpful for endemic/epidemic/pandemic diseases where there is an insufficient sense of urgency to drive the massive and decisive public intervention and investment to undergird corporate vaccine development.

P25 - Social injustice is certainly unsettling, and I appreciate the themes in this discussion, but focussing on challenge trials in this way seems a stretch. Short of global social revolution, what are the alternatives? Historically, of course, where there is sufficient potential for a market in high income countries, the alternative has been truly parasitic research, with new therapeutics tested cheaply in lower income countries where they would ultimately not be accessible. We have largely licensed development of drugs and vaccines to a small number of giant corporate entities. Giving go/no-go decision making to for-profit pharma giants introduces myriad ethical problems. The use of modern challenge trials to subvert some of the distortions this situation has introduced seems to be a fairly low-level ethical challenge, considered in the full context of global social injustice.

**Agreed. Edited out most of these paragraphs.**

'Risky research' is used a number of times in these final paragraphs, emphasising my earlier point that it might be worth discussing this term earlier in greater detail. Risky, for whom? Or is this just so obvious in an ethics journal that it is not required?

The first and second justice/equity points are really sequential rather than separate ideas, aren't they? The second point is literally 'a more just effort to develop Covid-19 vaccines'. The point here is that COVID-19 has become like many of the conditions discussed in the earlier paragraphs - without a really push from governments, big pharma is essentially 'done' with COVID-19, and have much more to gain from convincing high income countries to invest in expensive boosters and/or vaccines targeting variants than in technologies to bring the benefits of vaccines to the populations of lower income countries (let alone 'next generation' vaccines). Challenge trials may be one mechanism to accelerate the development of 'less commercial' candidates. Again though, much of this discussion revolves around HIC governments refusing to leverage their power in the public-private partnerships that delivered COVID-19 vaccines. I simply cannot over-emphasise or repeat too many times that massive public investment in corporate vaccine development was The real 'warp drive' for COVID-19 vaccine development. Without a global social revolution, it is almost impossible to imagine similar mobilisation of public investment beyond the extreme context of the early period of a deadly pandemic. Even now, as easy as is it to foresee another pandemic in the not-too-distant future, it seems likely that funding for pandemic preparedness will dry up once the pressures of this pandemic have passed in high income countries.

**Revised this section in accordance with Reviewer #1’s comments. That should address these concerns too.**

Again, thank you for the opportunity to review this manuscript. I hope some of my commentary offered above has been useful.
